# The Knowledge and Practice of Family Planning Among Muslim Women in Rural Karnataka, India

**DOI:** 10.7759/cureus.58088

**Published:** 2024-04-11

**Authors:** I Nasreen, Mohammed Guthigar, Irene Veigas

**Affiliations:** 1 Department of Social Work, Yenepoya (Deemed to be University), Mangaluru, IND

**Keywords:** karnataka, muslim women, family planning, practices, knowledge

## Abstract

Introduction

Women's health is significantly impacted by undesirable pregnancies and unsafe abortions, leading to the deaths of thousands of women and causing many others to suffer from infertility and long-term reproductive health issues. To address this problem, the use of modern contraception methods is increasing, regardless of economic status and dwelling, allowing women to exercise their rights and access reproductive health services.

Objective

The objective of this study is to examine the knowledge and practice of family planning and the factors associated with it.

Setting and design

A cross-sectional study was conducted among Muslim women (n=461) using a two-stage stratified random sampling method with a semi-structured questionnaire in the five taluks of Dakshina Kannada district in Karnataka.

Methods and materials

The required study sample was drawn by adopting the two-stage stratified random sampling technique. The study population was divided into different strata consisting of panchayats and wards. The number of households was selected from these panchayats/wards.

Statistical analysis

The association between faith in family planning and the knowledge and practice of family planning is studied using a chi-squared test. The factors associated with the practice of family planning are identified using a univariable and multivariable logistic regression analysis. Statistical analysis was performed using the Statistical Package for Social Sciences (SPSS) version 27 (IBM SPSS Statistics, Armonk, NY).

Results

There is a significant association between faith in family planning and its practice, with a p-value of less than 0.0001. Age was also found to be a significant factor associated with the practice of family planning. Specifically, women aged 31-40 were less likely to practice family planning compared to women aged 21-30, with a p-value of 0.012. The majority of individuals indicated the preferred child (23.9%) and going against the custom (16.5%) as their reasons for not using family planning.

Conclusions

Muslim women are reasonably well-informed about modern methods of contraception, but their usage remains relatively low. Outreach initiatives, health worker mobilization from within the community, and health education and information sharing are examples of program interventions that could help address this issue.

## Introduction

Globally, approximately 842 million women of reproductive age utilize modern methods of birth control, with 219 million relying on female sterilization. According to the World Family Planning 2022 report, the percentage of women using modern contraception increased from 35% in 1990 to 45% in 2021. The National Family Health Survey (2019-2021) indicates that 38% of women in India use contraceptives [1,2].

Women's health is profoundly affected by unwanted pregnancies and unsafe abortions, leading to the death of thousands and causing many to suffer from infertility and long-term reproductive health issues [3]. A study across 172 nations revealed that contraception prevents over 54 million unplanned births, unintended births, infant mortality, miscarriages, and maternal mortality deaths [4]. To address this, the use of modern contraception has risen, allowing women, regardless of economic status, to exercise their rights and access reproductive health services. While there is free availability of modern contraception in government clinics, the unmet need for contraception persists [5,6].

In 2019, the World Health Organization (WHO) initiated the Family Planning Accelerator Project to enhance and provide qualitative family planning services [7]. Despite the positive impact of family planning on preventing unintended pregnancies and maternal mortality, many women face an unmet need for contraception due to factors such as inadequate access, side effects, resistance from family, and the lack of awareness. The Sustainable Development Goals (SDG) aim to increase family planning action by addressing individual-level characteristics such as age, marital status, education, and media exposure [8-10].

The low status of women and the widespread preference for male progeny are the two most notable patriarchal constraints in India [11,12]. Family planning delays a young woman's first pregnancy when she may have premature pelvic development, reduces the likelihood of an unsafe abortion, and lowers the number of maternal deaths [13]. The usage of contraception has a long history in Islam, and opinions on its use vary among religious leaders [14-16]. Although Muslim women have adopted the usage of family planning, fertility rates remain higher, often attributed to lower levels of education [17-22].

Appropriate family planning can prevent both unsafe abortions and unwanted pregnancies. Modern contraceptives are valuable, but not everyone has access to or uses them [23]. Decisions about family planning have been directly impacted by an increase in the literacy rate, increased opportunities for women to pursue higher education and careers, and improved family socio-economic status [24].

## Materials and methods

Methodology

A cross-sectional study was undertaken among Muslim women in Dakshina Kannada, Karnataka, India. Before data collection, institutional ethical clearance was obtained from the Ethics Committee-1 of Yenepoya (Deemed to be University) with protocol number YEC-1/2018/235. Additionally, consent was sought from the participants before conducting direct interviews, utilizing a semi-structured questionnaire.

Sample size and sampling technique

The study focused on Muslim households in Dakshina Kannada, with a total of 461 women recruited as participants from five taluks: Mangaluru, Bantwal, Puttur, Belthangady, and Sullia. The two-stage stratified random sampling strategy was employed for household selection. Family lists were collected from each of the panchayats that formed the sampling frame. The study population was stratified based on panchayaths and wards. Rural and urban classifications were based on government records, where panchayath details were collected from the taluk panchayath office, and urban details were collected from the city/municipality. The number of households chosen for the study was determined from the selected panchayaths/wards.

Sampling method

**Figure 1:**
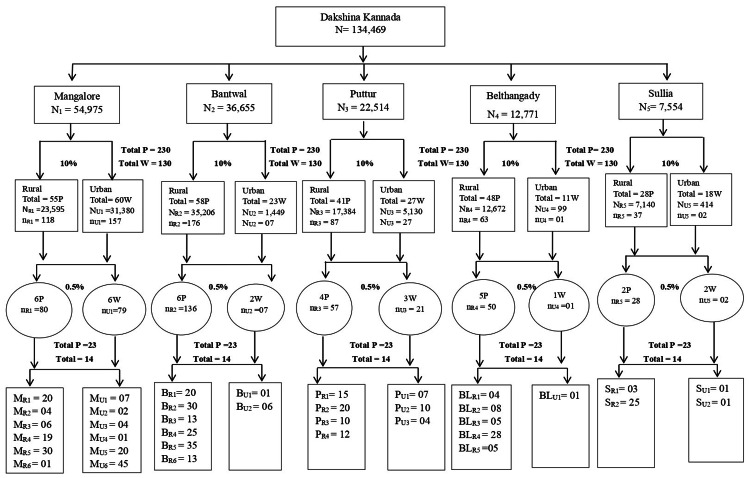
Detailed sample size estimation P, panchayath; W, ward; R, rural; U, urban; M, Mangaluru; B, Bantwal; P, Puttur; BL, Belthangady; S, Sullia; N, total number of households in a taluk; n, number of households selected from a taluk

From the five taluks of Dakshina Kannada, 23 panchayaths and 14 wards were selected. The primary unit is panchayath/ward, and the secondary unit is households. The first stage of the sampling unit was selected using a simple random sample, and the second stage of the sampling unit was selected using a systematic sampling method. The number of samples from each strata (primary unit) is determined using proportional allocation. Detailed sample size estimation is given in Figure 1.

The formula used for sample size estimation is as follows: in the first stage, panchayaths/wards were selected using simple random sampling without replacement (SRSWOR), with the criteria being 10% of the total panchayaths/wards. The allocation of the sample size for each stratum is given by nh=(n/N)Nh, where Nh denotes the total number of households in the selected strata, N denotes the total number of households in a taluk, n denotes the number of households to be selected from a taluk, and nh denotes the number of households to be selected from a strata (i.e., panchayaths/wards).

The second stage of the sampling unit was done using a systematic sampling method, and the calculated sampling length is k=Nh/nh.

Inclusion and exclusion criteria

The study included women aged between 21 and 50 years. The inclusion criteria encompassed women within this age range. However, specific exclusion criteria were applied, excluding widowed, divorced, and single women from participation in the study.

Data collection method

Data collection utilized a validated, semi-structured questionnaire designed to assess the participants' knowledge and practice of family planning. The questionnaire comprised two parts. The first part gathered socio-demographic details, including age, place of residence, marital status, education level, occupation, and income, categorized based on above poverty line (APL) and below poverty line (BPL) criteria. The second part focused on the participants' knowledge of family planning, contraceptive use, religious faith, practices, types of methods used, and reasons for not practicing family planning.

The questionnaire underwent testing, validation, and necessary adjustments. The data were collected from the participants covering the period from January to June 2023. After obtaining consent and providing participant information sheets, interviews were conducted in the local language: Kannada. The questionnaire was translated into Kannada for better comprehension.

Statistical analysis

The socio-demographic variables are summarized as frequency and percentage. The association between faith in family planning and the knowledge and practice of family planning is studied using a chi-squared test. The factors associated with the practice of family planning are identified using a univariable and multivariable logistic regression analysis. The reasons for not practicing family planning and the methods of family planning are summarized as frequency and percentage. A p-value of less than 0.05 is considered statistically significant. The data are analyzed using the Statistical Package for Social Sciences (SPSS) (version 27) software (IBM SPSS Statistics, Armonk, NY).

## Results

The study revealed that 42.5% of the participants belonged to the age group of 31-40 years. In terms of education, a majority (60.7%) had completed education up to the primary level. Regarding family structure, 74.0% of the participants were residing in nuclear families. Occupation-wise, 44.0% identified as homemakers, and 14.1% were engaged in beedi rolling work. In terms of monthly income, nearly 73.0% of the participant's families fell within the income range of ₹3,000-₹11,000 ($36-$60) (Table 1).

**Table 1:** Distribution of study participants based on their socio-demographic variables (n=461) *Students and incapacitated to work INR, Indian rupee; NA, not applicable

Variables	Categories	Frequencies (%)
Age (years)	21-30	79 (17.1)
	31-40	196 (42.5)
	41-50	186 (40.3)
Education level	≤Primary	280 (60.7)
	>Primary	181 (39.3)
Family type	Nuclear	341 (74.0)
	Joint	120 (26.0)
Occupation	Unemployed	118 (25.6)
	Daily wage worker	2 (0.4)
	Homemaker	203 (44.0)
	Beedi roller	65 (14.1)
	Government servant	4 (0.9)
	Self-employed	3 (0.7)
	NA*	66 (14.3)
Per capita income (INR)	<3,000	5 (1.1)
	3,001-5,000	201 (43.6)
	5,001-7,000	42 (9.1)
	7,001-9,000	28 (6.1)
	9,001-1,100	60 (13.0)
	>11,001	125 (27.1)

The majority of the participants (93.7%) had knowledge of family planning, and 77.7% of the participants did not practice family planning. Of the participants, 58.4% were not sure about family planning (Table 2).

**Table 2:** Distributions of study participants based on knowledge and practice of family planning (n=461) Data presentation: the data of the Muslim women are expressed as frequency (f) and percentage (%)

Serial number	Variable	Categories	Frequencies (%)
1	Knowledge of family planning	Yes	432 (93.7)
		No	29 (6.3)
2	Practice of family planning	Yes	103 (22.3)
		No	358 (77.7)
3	Faith in family planning	Yes	127 (27.5)
		No	65 (14.1)
		Not sure	269 (58.4)

A statistically significant association was not evident between the knowledge of family planning and its practice. The percentage of individuals practicing family planning with and without knowledge of it remains quite close, with 22.5% of those with knowledge and 20.7% without engaging in family planning. In contrast, faith in family planning demonstrates a significant association with its practice, with a p-value of less than 0.0001. Individuals who express faith in family planning are much more likely to engage in it, with 63% of believers practicing it compared to only 3.1% of nonbelievers. This highlights the powerful influence of faith or belief systems in shaping behaviors related to family planning (Table 3).

**Table 3:** Association between faith in family planning and the knowledge and practice of family planning (n=461) Test used: chi-squared (χ^2^) test

		Practice of family planning		Total (%)	Test statistics	p-value
		Yes (%)	No (%)			
Knowledge of family planning	Yes	97 (22.5)	335 (77.5)	432 (100.0)	0.049	0.825
	No	6 (20.7)	23 (79.3)	29 (100.0)		
Faith in family planning	Yes	80 (63.0)	47 (37.0)	127 (100.0)	167.61	<0.0001
	No	2 (3.1)	63 (96.9)	65 (100.0)		
	Not sure	21 (7.8)	248 (92.2)	269 (100.0)		

The univariable analysis is carried out by considering one variable at a time, and a variable that is significant at a 30% level is taken to multivariable analysis. Among the 461 respondents, 103 of them practiced family planning (22.5%). The age was found to be a significant factor associated with the practice of family planning. The women belonging to the 31-40 age group were less likely to practice when compared to women belonging to the 21-30 age group (Table 4).

**Table 4:** Factors associated with the practice of family planning (n=461) Test used: logistic regression

Variable	Category	Frequency	Percent	Univariable analysis		Multivariable analysis	
				Odds ratio	p-value	Odds ratio	p-value
Age	21-30	275	59.65	Reference		Reference	
	31-40	186	40.35	0.53	0.005	0.559	0.012
Location	Rural	333	72.23	Reference			
	Urban	128	27.77	1.038	0.881		
Educational level of the respondent	Primary and below	284	61.61	Reference			
	Above primary	177	38.39	1.209	0.415		
Family type	Nuclear	341	73.97	Reference			
	Joint	120	26.03	1.293	0.332		
Age at marriage	12-17	85	18.44	Reference		Reference	
	18-23	330	71.58	1.391	0.252	1.483	0.176
	Above 23	46	9.98	0.466	0.001	0.585	0.186
Occupation of the respondent	Unemployed	387	83.95	Reference			
	Employed	74	16.05	0.958	0.887		
Per capita income of family per month	≤5,000	5	1.08	Reference		Reference	
	>5,000	456	98.92	5.43	0.0069	3.504	0.207

The majority of the participants (77.7%) did not practice family planning, and 12.8% of them were practicing sterilization. The majority of the participants' reasons for not practicing family planning were the preferred child (23.9%) and against the custom (16.5%) (Table 5).

**Table 5:** Distributions of the study participants based on reasons for not practicing and types of family planning practiced (n=461) Data presentation: the data of the Muslim women are expressed as frequency (f) and percentage (%) *Permission from husband/permission from the family NA: not applicable

Variable	Categories	Frequencies (%)
Reason for not practicing family planning	Against our custom	76 (16.5)
	Not good for health	44 (9.5)
	Preferred child	110 (23.9)
	Cannot say	92 (20.0)
	Do not know	17 (3.7)
	Others*	17 (3.7)
	NA	105 (22.8)
Types of family planning methods practiced	Pills	28 (6.1)
	Loops	12 (2.6)
	Sterilization	59 (12.8)
	No opinion	4 (0.9)
	NA	358 (77.7)

## Discussion

This study delves into the knowledge and utilization of family planning among Muslim women. The findings indicate that a significant proportion of Muslim women exhibit suboptimal contraceptive practices despite possessing a relatively good understanding of modern contraceptive methods. This aligns with the results of a study conducted by Sharma and Pasha, providing consistency in the observed patterns [25]. Notably, contraceptive usage was low among women with educational attainment below the primary level and those residing in nuclear families. The latter group's limited exposure within nuclear family settings may contribute to a restricted awareness of contraceptive options. These findings resonate with a study conducted in India by Wani et al. [26] and Mishra [27].

Furthermore, the study reveals that modern contraceptive utilization is less prevalent among specific subgroups, such as homemakers with an income ranging between ₹3,001 and ₹5,000 ($36-$60), women who are married, and those married at the age of 18-23 years. This correlation aligns with the findings of Thakuri et al. [8]. The identified associations highlight the intricate interplay of socio-economic factors, marital status, and age at marriage in influencing contraceptive practices among Muslim women.

This study, in conjunction with Sharma et al.'s [16] research, highlights the knowledge levels among urban and rural women regarding family planning and contraceptive methods. Drawing parallels with Shumayla and Kapoor's [21] findings, approximately 87% of Indian women were aware of modern contraceptive methods, while Dhakal et al.'s [17] study reported a higher knowledge rate of 94.5%. Notably, the present study underscores the positive impact of women's education on enhancing knowledge of family planning techniques, although it also reveals a decrease in the actual practice of family planning. In contrast, Mohanan et al.'s [28] research found that monthly income significantly influenced women's acceptance of family planning methods, with no significant impact observed for educational attainment.

Concerning Srivastava et al.'s research [29], this study aligns with the widespread awareness of female sterilization, yet it underscores a significant lack of knowledge regarding temporary family planning methods. In contrast to the results obtained by Rao et al. [30], the present study observed that 46.9% of women were aware of sterilization, followed by pills (27.5%), condoms (7.2%), and intrauterine contraceptive devices (IUCD) (8.5%).

In comparing the current study with the results presented by Mohanan et al. [28] and Boonstra [14], it is evident that, despite having a comprehensive knowledge of modern contraceptive methods, Muslim women display reluctance toward family planning. This resistance can be traced back to factors such as adherence to cultural norms, specific preferences regarding the number of children, and apprehensions about potential health implications. The nuanced insights derived from these comparisons underscore the necessity for culturally tailored and community-specific strategies in implementing family planning initiatives among Muslim women.

Relevance and recommendations

Family planning serves as a cornerstone of reproductive health, empowering individuals to make informed decisions regarding pregnancy timing and spacing. Within rural Karnataka, particularly among Muslim women, distinct cultural, religious, and socio-economic factors significantly influence family planning practices. Understanding the context-specific relevance of family planning is pivotal for crafting effective strategies aimed at enhancing reproductive health and overall well-being. The present study examines the knowledge and practices of family planning among Muslim women in rural Karnataka, India, and sheds light on the existing gaps within religious minority communities. This investigation delves into the intersection of family planning knowledge and practices within the framework of Islam, acknowledging diverse opinions and religious barriers associated with contraception. Such cultural sensitivity is paramount in designing interventions that honor and resonate with the values and beliefs of the Muslim community, necessitating a call to action to review existing policies and programs geared toward promoting family planning practices among Muslims in Karnataka.

Given the relatively low adoption rates of modern contraception methods among Muslim women, the study advocates for targeted outreach initiatives tailored to address the unique challenges and cultural nuances within the Muslim community. Mobilizing health workers from within the Muslim community can serve as a linchpin in bridging the knowledge-practice gap. Culturally competent health workers are poised to act as trusted sources of information, addressing concerns and delivering personalized guidance on family planning. This approach facilitates informed decision-making and fosters the increased utilization of modern contraceptive methods. Engaging the Muslim community in the co-creation and implementation of family planning interventions is imperative. Involving community leaders, religious figures, and influencers can foster understanding, dispel myths, and encourage open dialogue surrounding family planning. The study advocates for future investigations to delve deeper into the variables contributing to resistance and barriers to family planning within the Muslim community. Exploring factors such as cultural customs, preferences, and health concerns can inform more targeted and effective interventions.

Strengths and limitations

The study boasts a robust methodology, employing a cross-sectional design and a two-stage stratified random sampling technique. This approach enhances the study's external validity, allowing for findings to be generalized to the broader population of Muslim women in rural Karnataka. The inclusion of a diverse sample from five taluks further bolsters the study's representativeness, capturing variations within the community. Additionally, the utilization of a semi-structured questionnaire, tested for validity, ensures comprehensive data collection, encompassing socio-demographic details, knowledge levels, and practices related to family planning. The study's adherence to ethical standards, including obtaining institutional ethical clearance and participant consent, underscores its commitment to ethical research practices.

Despite its strengths, the study bears certain limitations that merit consideration. The cross-sectional design, while valuable for snapshot insights, limits the establishment of causal relationships and temporal sequences. Reliance on self-reported data introduces the potential for recall bias and social desirability bias, impacting the accuracy of responses. Furthermore, the study's focus on rural Karnataka may not fully encapsulate the diversity of experiences among Muslim women in urban settings. Nevertheless, the study contributes valuable insights and lays a foundation for future research and targeted interventions.

## Conclusions

In conclusion, the study on family planning among Muslim women in rural Karnataka not only highlights the current landscape but also provides valuable insights for actionable recommendations. By addressing the identified challenges through culturally sensitive and community-specific interventions, there is a potential to positively impact family planning practices and, consequently, the reproductive health outcomes of Muslim women in the region.
